# Sexual Chemosignals: Evidence that Men Process Olfactory Signals of Women’s Sexual Arousal

**DOI:** 10.1007/s10508-019-01588-8

**Published:** 2020-02-05

**Authors:** Arnaud Wisman, Ilan Shrira

**Affiliations:** 1 School of Psychology, Keynes College, University of Kent, Canterbury, Kent CT2 7NP UK; 2 Department of Psychology, Pennsylvania State University, University Park, PA USA

**Keywords:** Sexual arousal, Chemosignals, Olfaction, Mating strategies, Gender

## Abstract

Research suggests that humans can communicate emotional states (e.g., fear, sadness) via chemosignals. However, thus far little is known about whether sexual arousal can also be conveyed through chemosignals and how these signals might influence the receiver. In three experiments, and a subsequent mini meta-analysis, support was found for the hypothesis that men can process the scent of sexually aroused women and that exposure to these sexual chemosignals affect the subsequent perceptions and sexual motivation of men. Specifically, Experiment 1 revealed that men evaluate the axillary sweat of sexually aroused women as more attractive, compared to the scent of the same women when not sexually aroused. In addition, Experiment 2 showed that exposure to sexual chemosignals increased the men’s sexual arousal. Experiment 3 found support for the thesis that exposure to sexual chemosignals would increase sexual motivation. As predicted, men devoted greater attention to and showed greater interest in mating with women who displayed sexual cues (e.g., scantily dressed, in seductive poses). By contrast, exposure to the sexual chemosignals did not alter males’ attention and mating interest toward women who displayed no sexual cues. It is discussed how sexual chemosignals may function as an additional channel in the communication of sexual interest and how contextual factors can influence the dynamics of human sexual communication.

## Introduction

An accumulating body of research suggests that humans, like other animals, can communicate information by means of olfactory signals. Specifically, scents released by the body have been shown to convey fitness-relevant information about a person’s physical health, fertility, and genetic relatedness, as well as emotional states such as fear (de Groot, Smeets, Kaldewaij, Duijndam, & Semin, 2012; Pankevich, Baum, & Cherry, 2004; Ziegler, Kentenich, & Uchanska-Ziegler, 2005). The release of chemosignals during emotional experiences can function as an additional channel of communication along with other modalities (e.g., visual, auditory) and prompt nearby perceivers to respond in adaptive ways (de Groot et al., 2012). For example, fearful or anxious experiences cause people to release body sweat that activates threat management responses in others (e.g., a stronger startle response, heightened vigilance), enabling conspecifics to respond to potential threats in ways that improve their likelihood of survival (de Groot et al., 2012).

In this paper, the possibility is explored that female sexual arousal leads to the release of an axillary chemosignal that can be detected by men, leading to increased sexual interest and sexual arousal. Although traditionally not defined as a basic emotion, sexual arousal has many of the same hallmarks of an emotion: It is a short-lived motivational–affective state elicited by particular stimuli, with an interrelated system between its physiology and expression (Ekman, 1984; Everaerd, 1989; Geer, Lapour, & Jackson, 1993). Moreover, the expression of sexual arousal serves important fitness-related functions in the signaling and coordinating of mating (Metts, Sprecher, & Regan, 1998). However, thus far there is only limited evidence that sexual arousal produces chemosignals that can be detected by the opposite sex.

In one study, for example, the axillary sweat of sexually aroused and non-aroused men was presented to women while monitoring the women’s brain activity (Zhou & Chen, 2008). The results showed that sweat released while the men were sexually aroused activated neural substrates involved in the processing of sexual stimuli (the hypothalamus) and socio-emotional significance (the fusiform area and orbitofrontal cortex) in the female recipients (Brunetti et al., 2008; Savic, Berglund, Gulyas, & Roland, 2001; Vuilleumier & Pourtois, 2007). Zhou and Chen (2008) concluded that the experience of sexual arousal caused males to release sweat that contained a unique, emotion-laden chemosignal related to sexual signaling. The present studies sought to test whether men could likewise be influenced by the scent of sexually aroused women and whether these sexual chemosignals would affect the subsequent perceptions and sexual motivation of men.

There are substantial theoretical and empirical grounds to expect that males should be sensitive to cues of female sexual arousal (Haselton & Buss, 2000; Trivers, 1972). As sexual strategies theory and error management theory have outlined (Buss & Schmitt, 1993; Haselton & Buss, 2000), the differential risks and benefits of sexual encounters for men and women lead men to assume a more opportunistic mating strategy, whereas women more often assume the gatekeeper role in choosing when and with whom to mate. As a result, men are afforded fewer mating opportunities and therefore bear a greater cost when these opportunities are missed. In support of these assumptions, studies have shown that men are more willing to have casual sex with strangers (Buss & Schmitt, 1993; Clark & Hatfield, 1989; Herold & Mewhinney, 1993; Oliver & Hyde, 1993), take more risks in consummating sexual opportunities (Ariely & Loewenstein, 2006), overestimate women’s sexual interest (Grammer, Kruck, Juette, & Fink, 2000), desire a greater number of sexual partners (Dewsbury, 1981; Wilson, Kuehn, & Beach, 1963), and lower their standards toward potential mates when sexual opportunities arise (Pennebaker et al., 1979; Szepsenwol, Mikulincer, & Birnbaum, 2013; for a broad review, see Baumeister, Catanese, & Vohs, 2001). Thus, the literature highlights that men are more responsive to sexual cues, more motivated to pursue mating prospects, and show cognitive biases aimed at avoiding missed sexual opportunities (Haselton & Buss, 2000). These findings suggest that men may be sensitive to chemosensory cues of women’s sexual arousal.

In fact, research has shown that men can detect (albeit outside conscious awareness) chemosensory cues related to female emotions and reproduction (Cerda-Molina, Hernández-López, Borráz-León, & Chavira-Ramírez, 2014; Gelstein et al., 2011; Haselton & Gildersleeve, 2011; Kuukasjärvi et al., 2004). For example, some research has shown that certain chemosensory cues can alter men’s sexual interest (Gelstein et al., 2011). Specifically, Gelstein et al. found that olfactory exposure to women’s tears (compared to a saline solution) diminished men’s self-reported and physiological sexual arousal, and led them to evaluate pictures of women’s faces as less sexually appealing. Another line or research suggests that men can distinguish between the scent samples of women who are high vs. low in fertility (Gildersleeve, Haselton, Larson, Pillsworth, 2012). Specifically, the scent of ovulating (vs. non-ovulating) women is evaluated by men as more attractive and pleasant (Singh & Bronstad, 2001; Tarín & Gómez-Piquer, 2002), increases men’s testosterone and cortisol levels (Cerda-Molina, Hernández-López, Claudio, Chavira-Ramírez, & Mondragón-Ceballos, 2013; Miller & Maner, 2009), increases the accessibility of sexual-related thoughts (Miller & Maner, 2011), increases their interest in sex (Cerda-Molina et al., 2013), and prompts men to engage in greater behavioral mimicry in subsequent interactions with a woman (Miller & Maner, 2011).

It is important to note, though, that while ovulatory timing can influence a woman’s sexual arousal in some contexts and toward certain types of mates, the fertile (follicular) phase culminating in ovulation is by no means a state of heightened chronic sexual arousal (Bossio, Suschinsky, Puts, & Chivers, 2014; Pillsworth, Haselton, & Buss, 2004; Slob, Bax, Hop, & Rowland, 1996). Moreover, the endocrinological changes that accompany the rise in fertility differ markedly from those associated with sexual arousal and sexual desire in women (Shirazi, Bossio, Puts, & Chivers, 2018; van Anders, Hamilton, Schmidt, & Watson, 2007; Vitzhum, 2009). For example, increased fertility is associated with elevated levels of progesterone and luteinizing hormone that can persist for days (Vitzhum, 2009), whereas sexual arousal is accompanied by short-term changes in hormones such as testosterone (Goldey & van Anders, 2011). Thus, it is unlikely that elevated fertility likelihood and sexual arousal produce a common olfactory output.

### Overview and Hypotheses

In three experiments, axillary perspiration samples were collected from female donors while they were sexually aroused and (at a different time) while they were non-sexually aroused; then, male recipients were exposed to each of these scent samples. Experiment 1 tested the hypothesis that males would evaluate the scent samples of sexually aroused females as more attractive than the non-sexual scents. Experiment 2 tested whether exposure to the sexual scent samples would increase males’ sexual arousal. Finally, Experiment 3 explored whether female sexual chemosignals augmented men’s attention to female sexual cues in a subsequent task. Specifically, in Experiment 3 it was examined whether exposure to sexual arousal scents would lead males to spend more time viewing photographs of scantily dressed women in seductive poses and report a greater motivation to pursue these women.

## Experiment 1

### Method

#### Female Scent Donors

Eleven heterosexual female students (*M* = 19.64, SD = 1.63 years) who were not on chemical contraception (Fleischman, Navarrete, & Fessler, 2010; Renfro & Hoffmann, 2013; Roberts, Cobey, Klapilová, & Havlíček, 2013) served as scent donors in a randomized double-blind experiment using a within-subjects design. Similar to previous research (e.g., Lenochova, Roberts, & Havlicek, 2009), scent donors in all of the experiments were instructed to avoid the following activities for 48 h prior to the study: smoking, drinking alcohol, eating spicy foods or garlic, using deodorant or perfume, or engaging in sexual activity.

#### Female Scent Sample Collection

After arriving at the laboratory and receiving a brief introduction to the study, participants were asked by a female experimenter to clean their underarms using fragrance-free wipes (e.g., de Groot, Smeets, & Semin, 2015b; Elliot, Muir, & de Catanzaro, 2017). Large cotton pads were then affixed to their underarms using surgical tape, and they were given a new white T-shirt to wear for the experiment.

Participants then completed one of the two conditions (watching either the sexual or neutral video) and returned 7 days later to serve in the other condition. The order of the conditions was randomized. Regardless of condition, all women were first asked to cycle on a stationary bicycle for 3 min at high (80%) intensity. This procedure was used because general physical arousal can produce similar endocrinological reactions as sexual arousal (e.g., elevated testosterone; Zitzmann & Nieschlag, 2001). Thus, including the exercise task helped to ensure that sexual arousal, rather than general physical arousal, would be the key difference between the two conditions (Dalton, Mauté, Jaén, & Wilson, 2013; Mujica-Parodi et al., 2009; Zernecke et al., 2011). After the women cycled for 3 min, they were seated in front of a 17-inch computer screen and watched a video for 20 min. In the neutral video condition, participants watched a clip from a documentary about bridge building. In the sexual arousal condition, participants watched a clip of the erotic cult film *9 Songs* (2004) that portrayed a series of explicit sex scenes between a man and a woman.

After watching the video, a manipulation check asked participants to answer two questions about their subjective sexual arousal: “To what extent were you sexually aroused whilst watching the clip?” (1 = not at all to 9 = *very much*) and “To what extent were you sexual stimulated by the clip?” (1 = *not at all* to 9 = *very much*). In addition, participants responded to two questions about how they felt while viewing the video (“To what extent did you feel positive whilst watching the clip?” (1 = *not at all* to 9 = *very much*) and “To what extent did you feel negative whilst watching the clip?” (1 = *not at all* and 9 = *very much*)), as well as a question that measured their interest in the video (“To what extent were you interested in the clip you were watching?”; 1 = *not at all* to 9 = *very much*). Finally, participants filled out a demographic questionnaire.

The cotton pads were then removed from their underarms, cut into four equal pieces, sealed in separate plastic bags (each with a unique ID code), and stored in a freezer (-25 °C) (Lenochova et al., 2009). At the conclusion of the second of the two sessions, everyone was fully debriefed and paid for their participation.

#### Male Scent Recipients

Twenty-four heterosexual male students (*M* = 21.38, SD = 3.49) were recruited for a study about evaluating female scents, with prerequisites that these men were not suffering from a blocked nose and should not wear any cologne on the day of the study.^1^ Each male participant was exposed to all 22 scent samples (both scents from each woman) in a randomized order and evaluated each scent on several dimensions (see below).

### Procedure

The female sweat samples were kept frozen until the next stage of the experiment approximately 1 week after all the female sweat samples were collected, at which point the cotton pads were defrosted and presented to males in small opaque airtight glass containers. Male recipients were informed that they would smell and evaluate samples that contained female body scents (one quarter of a pad per container; each quarter of a pad was discarded after each laboratory day). They then rated each scent sample on three dimensions: intensity (1 = *not at all intense* and 7 = *very intense*), pleasantness (1 = *very unpleasant* and 7 = *very pleasant*), and sexiness (1 = *not at all sexy* and 7 = *very sexy*). Following previous research (Thornhill & Gangestad, 1999), the pleasantness and sexiness ratings were averaged into a total “attractiveness” index (the two items were internally consistent, *α* = .83). After rating all the samples, males completed a demographic questionnaire. Participants were then fully debriefed and paid for their participation.

### Results and Discussion

#### Female Scent Donors: Manipulation Checks and Perception of the Video

We began by verifying that the video stimulus was effective in manipulating females’ sexual arousal. The manipulation checks revealed that they were: Women reported greater sexual arousal while watching the sexual content video (*M* = 4.64, SD= 2.46) relative to the neutral video about bridge building (*M* = 1.55, SD = .82), *F*(1, 10) = 15.25, *p* = .003, *η*^*2*^ = .60. Similarly, women viewing the sexual content video reported being more sexually stimulated (*M* = 4.36, SD= 2.34) than while watching the neutral video (*M* = 1.27, SD = .65), *F*(1, 10) = 15.71, *p* = .003, *η*^*2*^ = .61. Furthermore, no difference was found between the two conditions in terms of positive affect (*F*[1, 10] = .51, *p* = .49), negative affect (*F*[1, 10] = .43, *p* = .53), or interest (*F*[1, 10] = .19, *p* = .67).

#### Male Recipients: Intensity Ratings of the Scent Samples

A one-way repeated-measures ANOVA was used to compare the average intensity ratings of the sexually arousal and neutral sweat samples. This analysis revealed that males rated the scents from the sexual arousal condition (*M* = 3.73, SD = .89) as equally intense as the scents from the neutral condition (*M* = 3.80, SD = .97), *F*(1, 23) = .27, *p* = .61.

#### Male Recipients: Attractiveness Ratings of the Scent Samples

The main hypothesis was that males would evaluate the sweat of sexually aroused females as more attractive than their non-sexual sweat. The findings confirmed this hypothesis: The sexual arousal scents were perceived as more attractive (*M* = 3.60, SD = 0.66) than the scents from the neutral condition (*M* = 3.33, SD = 0.51), *F*(1, 23) = 7.98, *p* = .010, *η*^*2*^ = .26.

Encouraged by these initial findings, Experiment 2 was designed with two goals in mind: to replicate the results of Experiment 1 and to explore the possibility that exposure to the female sexual scent samples would influence the subsequent sexual arousal of the male recipients. One consistent theme of the findings on emotion chemosignals (e.g., fear) is that they produce emotional contagion. That is, upon exposure to these chemosignals, recipients tend to simulate the same emotion experienced by the donor (Semin & de Groot, 2013). Contagion effects can serve to tune recipients to emotionally relevant cues (e.g., threats, opportunities) that guide adaptive behavioral responses (e.g., avoidance, approach) to stimuli in the environment (de Groot et al., 2012). Thus, it was expected that when exposed to olfactory signals of female sexual arousal, males would respond with increased sexual arousal themselves.

## Experiment 2

### Method

#### Female Scent Donors

Six heterosexual female students (*M* = 19.33, SD = .52) who were not on chemical contraception served as scent donors in a double-blind within-subjects experiment.^2^ In Experiment 2, the procedures, stimulus videos, and questions for the female scent donors were nearly identical to those in Experiment 1. The only difference was that in Experiment 2, women rated the stimulus videos on one additional dimension (boredom) with this question: “To what extent did you find the video boring?” (1 = *not at all* to 9 = *very much*).

#### Male Scent Recipients

Thirty-two heterosexual male students (*M* = 21.44, SD = 2.48) served as scent recipients, again with the prerequisites that they were not suffering from a blocked nose and not wearing any cologne on the day of their session.

### Procedure

The procedures and scent evaluation questions for the males in Experiment 2 were slightly different from those in Experiment 1. Most of these changes were designed to test whether priming males with either the sexual or neutral female scent samples would alter their own sexual arousal. To do this, the scent evaluation task consisted of two parts, which the males completed in a randomized order. One part had males rate the six sexual scent samples, and the other part had them rate the six neutral scent samples. Participants were instructed to relax for 3 min between each scent block (de Groot et al., 2015a). Each scent sample (also presented in randomized order within each block of six) was evaluated on four dimensions: intensity (1 = *not at all* and 7 = *very much*), pleasantness (1 = *not at all* and 7 = *very much*), sexiness (1 = *not at all* and 7 = *very much*), and attractiveness (1 = *not at all* and 7 = *very much*). The latter dimension was added to Experiment 2 to improve the construct validity of the perceived attractiveness dependent variable. The overall “attractiveness” index for both the sexual samples and the neutral samples was created by averaging the three ratings for attractiveness, pleasantness, and sexiness (*α* = .91 for the sexual scent samples; *α* = .90 for the neutral scent samples).

To test whether exposure to the scents affected men’s own subjective sexual arousal, after completing each scent evaluation block (evaluating each set of six scent samples), they were asked “To what extent do you feel sexually aroused right now?” (1 = *not at all* and 7 = *very much*).

Like Experiment 1, males then filled out a short demographic questionnaire. After that, they were fully debriefed and paid for their participation.

### Results and Discussion

#### Female Scent Donors: Manipulation Checks

Female scent donors watching the sexual content video reported greater sexual arousal (*M* = 6.33, SD= 1.51) than while watching the neutral video (*M* = 1.00, SD = .00), *F*(1, 5) = 75.29, *p* < .001, *η*^*2*^ = .94. Similarly, those viewing the sexual content video reported being more sexually stimulated (*M* = 5.67, SD= 2.16) than when they watched the neutral video (*M* = 1.00, SD = .00), *F*(1, 5) = 28.00, *p* = .003, *η*^*2*^ = .85. Unexpectedly, it was found that women reported greater negative affect while watching the sexual video (*M* = 2.83, SD= .98) compared to the neutral video (*M* = 1.67, SD = .52), *F*(1, 5) = 8.45, *p* = .034. No difference was found between the two conditions in terms of positive affect (*F*[1, 5] = 2.50, *p* = .18), interest (*F*[1, 5] = 1.36, *p* = .30, or boredom (*F*[1, 5] = 2.50, *p* = .18).

#### Male Recipients: Intensity Ratings of the Scent Samples

As in Experiment 1, a repeated-measures ANOVA revealed that male recipients rated the female scent samples from the sexual arousal condition (*M* = 2.41, SD = .74) as equally intense as those in the neutral condition (*M* = 2.29, SD = .69), *F*(1, 31) = 1.19, *p* = .29.

#### Male Recipients: Attractiveness Ratings of the Scent Samples

Consistent with our hypothesis, and Experiment 1, the scent samples collected from women who watched the sexual video were evaluated by men as more attractive (*M* = 2.44, SD = .83) compared to the scents collected during the neutral video (*M* = 2.18, SD = .76), *F*(1, 31) = 4.42, *p* = .044, *η*^*2*^ = .13.

#### Male Recipients: Sexual Arousal as a Function of Condition

**Fig. 1:**
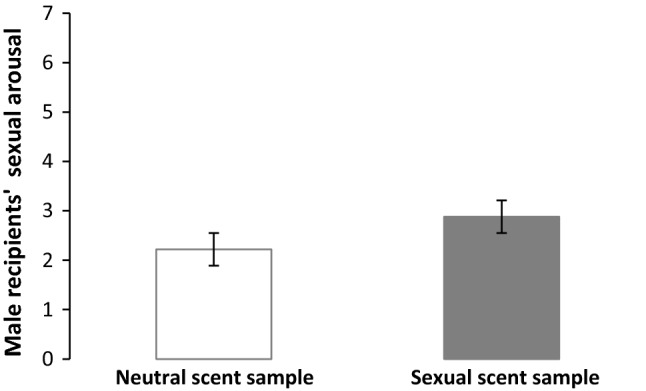
Male recipients’ sexual arousal after exposure to female scent samples from both conditions (Experiment 2). *Note* Error bars represent 95% confidence intervals

Finally, we examined whether the female scent condition would influence the sexual arousal among males. Consistent with the primary hypothesis, men reported greater sexual arousal after exposure to the scents of women watching the sexual video (*M* = 2.88, SD = 2.14) compared to their scents while watching the neutral video (*M* = 2.22, SD = 1.88), *F*(1, 31) = 8.03, *p* = .008, *η*^*2*^ = .21 (see Fig. 1).^3^

Experiment 2 replicated the scent rating findings from Experiment 1. Moreover, the finding also revealed that exposure to the women’s sexual scent samples heightened men’s sexual arousal, consistent with prior work showing emotional contagion of the sender’s affective state (Semin & de Groot, 2013). In this case, a contagion effect could help orient the recipient to the sender and increase the recipient’s motivation to respond to this sexual message.

Experiment 2 also found that females viewing the sexual stimuli reported greater negative affect (though no difference was found in positive affect) than when watching the neutral video. However, women’s level of self-reported sexual arousal was not correlated with their negative or positive affect (both *p*s > .5), and thus, it seems unlikely that negative affect influenced the women’s experience of sexual arousal. Experiment 3 sought to extend the main findings from Experiment 2.

Increased sexual arousal amplifies sexual motivation, and as a result, can influence thoughts and behavior in a variety of ways (Ariely & Loewenstein, 2006). For example, heightened sexual arousal causes males to show a greater focus on sexually relevant cues in possible mates (Pfaus, 1999) and an increased motivation to go on a date with them (Greitemeyer, 2005; Seal, Agostinelli, & Hannett, 1994; van Straaten, Engels, Finkenauer, & Holland, 2008). Greater sexual arousal also leads males to overestimate sexual opportunities (e.g., perceiving females as more interested), to be less discriminating about (and discount) the attractiveness of potential sexual partners (Baumeister et al., 2001) and to be more attentive to sexual cues (Baumeister et al., 2001; Confer, Perilloux, & Buss, 2010). Given that the scent of sexually aroused women increased men’s own sexual arousal (Experiment 2), Experiment 3 explored whether exposure to these scents would also increase men’s attention to sexual cues and interest in women in a subsequent task. This possibility was tested by exposing men to photographs of women with varying degrees of sexually salient cues. In turn, if exposure to sexual chemosignals increases men’s sexual arousal, it should also motivate them to view photographs with a higher degree of sexual cues for longer periods of time and to express a greater desire to mate with the depicted women (described in detail below). By contrast, we did not expect that exposure to sexual chemosignals would affect men’s interest in women who displayed no overt sexual cues.

## Experiment 3

### Method

#### Female Scent Donors

Seven female students (*M* = 19.71, SD = 1.43) who were not taking chemical contraception served as scent donors in an experimental design similar to the first two studies.

#### Scent Sample Collection

The procedures for Experiment 3 scent collection were similar to the previous studies, except for the stimuli used while collecting the sweat samples from the women. In this experiment, several different types of stimuli were used in an attempt to strengthen the manipulation and test whether sexual arousal induction would generalize beyond the video. Rather than watching only a 20-min video, participants were exposed to four separate stimuli (containing either all sexual or all neutral content): a short video clip (approximately 2 min long), a short story (approximately 700 words), an excerpt from a long video clip (approximately 20 min long), and 20 images.

In the sexual content condition, the short video clip portrayed an erotic male dance performance taken from the movie “Magic Mike.” The short story was an excerpt taken from the novel *50 Shades of Grey* (James, 2012). Similar to Experiments 1 and 2, participants then watched the 20-min clip form the movie “9 Songs.” Finally, the images were 20 pictures taken from International Affective Picture System database (Lang, Bradley, & Cuthbert, 2005), some of which depicted opposite-sex couples engaged in a sexual act and the others depicting images of nude males.

In the neutral content condition, participants watched a short video clip that portrayed (non-sexual) contemporary dancing by men and women, read a story about knitting, watched the 20-min neutral clip about bridge building, and viewed 20 images depicting tropical birds.

Each prime (the text, videos, and images) was followed by a short set of questions: “To what extent where you sexually aroused whilst reading the text/watching the video/viewing these pictures?” (1 = *not at all* to 7 = *very much*), “To what extent were you interested in the text you read/video you watched/pictures you viewed?” (1 = *not at all* to 7 = *very much*), “To what extent did you feel positive whilst reading the text/watching the video/viewing the pictures?” (1 = *not at all* to 7 = *very much*), “To what extent did you feel negative whilst reading the text watching the video/viewing these pictures?” (1 = *not at all* to 7 = *very much*), and “To what extent did you find the text/the video/these images boring?” (1 = *not at all* to 7 = *very much*).

Like Experiments 1 and 2, at the end of each session, the experimenter removed the cotton pads from participants’ underarms, cut each cotton pad into four even pieces and placed them in two separate plastic bags, which were then tightly sealed, assigned a unique ID code, and kept frozen (− 25 °C). Participants then answered a demographics questionnaire. After completing both conditions, participants were fully debriefed and paid for their contribution.

#### Male Scent Recipients

Thirty-five heterosexual male students (*M* = 23.11, SD = 7.12) participated in a study advertised as being about evaluating scents. The prerequisites for participation by males were the same as in Experiments 1 and 2.

### Procedure

Experiment 3 had male recipients to complete scent ratings followed by a picture evaluation task and then do so again for the second scent condition. Similar to Experiment 2, the scent evaluation task entailed rating the scent samples in two separate blocks (again, with a 3-min break between the blocks): the seven scent samples from the sexual arousal condition and the seven from the neutral condition. (The two blocks were given in randomized order, with the individual scents randomized within each block.) Each individual scent was again rated on four dimensions: intensity (1 = *not at all* and 7 = *very much*), pleasantness (1 = *not at all* and 7 = *very much*), attractiveness (1 = *not at all* and 7 = *very much*), and sexiness (1 = *not at all* and 7 = *very much*). Like Experiment 2, the perceived “attractiveness” of the scents was created by averaging the three ratings for attractiveness, pleasantness, and sexiness (*α* = .94 among the sexual scent samples; *α* = .94 among the neutral scent samples).

After each scent evaluation block, the men were asked to evaluate 20 pretested casual photographs of different target women who were in their early to mid-twenties (Thomas & Stewart-Williams, 2018), and the distribution of ethnicities among the women was equivalent in each of the groups.^4^ Half of these pictures showed women who were dressed revealingly and stood or sat in provocative poses (high sexual salience). The other half of the pictures showed women who were modestly dressed and stood or sat in neutral poses (low sexual salience). Importantly, the pilot study (see Footnote 4) revealed that participants evaluated the promiscuous group as dressed more revealingly and higher in their perceived receptivity to sexual offers. As a shorthand, these two groups of stimulus photographs are referred to as *promiscuous* and *modest* targets.

After male recipients completed each scent evaluation block (i.e., the sexual or neutral scent samples), they completed the picture evaluation task that consisted of ten pictures: Five of the promiscuous targets and five of the modest targets were randomly chosen from the larger pool of the 20 stimulus photographs, and these 10 photographs were presented in a randomized order. The target photographs were displayed on a computer screen one at a time, and participants had to click to advance to the next screen when they were finished viewing the photograph. Each target photograph was displayed for a maximum of 10 s. On the subsequent screen, males were asked to rate the target’s attractiveness (“How attractive is this person?”; 1 = *not at all* and 10 = *very much*), to what extent they wanted to go on a date with the target (“Would you like to go on a date with this person?”; 1 = *not at all* and 10 = *very much*), and to what extent they desired a relationship with the target (“Would you like a relationship with this person?”; 1 = *not at all* and 10 = *very much*).

Experiment 3 employed two indices of men’s sexual interest in the target women (see Brown, Young, Sacco, Bernstein, & Claypool, 2009; Quinsey, Ketsetzis, Earls, & Karamanoukian, 1996; Rule, Rosen, Slepian, & Ambady, 2011; Rupp & Wallen, 2009). The first index was based on how much time males spent viewing each of the two groups’ photographs (promiscuous vs. modest target photographs). This value was created for each participant by subtracting the amount of time (in seconds) they spent viewing the promiscuous targets from the time they spent viewing the modest targets, whereby higher scores indicated relatively longer viewing times of the promiscuous targets. Thus, higher values denoted greater interest in the promiscuous targets. The second index of sexual interest was the extent to which males expressed a desire to mate with the targets, which was created by averaging the men’s ratings of the target’s perceived attractiveness, their interest in going on a date, and their interest in a relationship with the target (all three items showed factor loadings higher than .85 and overall high reliability *α*s > .94; see Brown et al., 2009; Gillath, Bahns, & Burghart, 2017).

After rating the ten photographs, the males completed the second block of scent ratings (for the other scent condition), after which they evaluated the remaining ten target women (the remaining five promiscuous and five modest targets, ordered randomly). Finally, participants filled out a short demographic questionnaire were fully debriefed and paid for their participation.

### Results

#### Female Scent Donors: Manipulation Checks

To simplify the reporting of the manipulation check results, the ratings of the four different types of stimulus content were averaged and evaluated by each female scent donor (i.e., short video, short story, long video, images), and did so along each of the dimensions (e.g., self-reported sexual arousal, interest). Women exposed to the sexual content reported greater sexual arousal (*M* = 4.71, SD= 0.48) than when exposed to the neutral content (*M* = 1.36, SD = 1.13), *F*(1, 6) = 165.68, *p* < .001, *η*^*2*^ = .96. No significant difference was found between the two conditions in terms of women’s interest (*F*[1, 6] = 2.25, *p* = .18), boredom (*F*[1, 6] = 4.23, *p* = .09), positive affect (*F*[1, 6] = 0.01, *p* = .92), or negative affect (*F*[1, 6] = 0.51, *p* = .50).

#### Male Recipients: Intensity Ratings of the Scent Samples

Male recipients rated the female scent samples from the sexual content condition (*M* = 3.42, SD = 1.31) as equally intense as the scents of women watching the neutral content (*M* = 3.37, SD = 1.33), *F*(1, 34) = 0.09, *p* = .76.

#### Male Recipients: Attractiveness Ratings of the Scent Samples

Experiment 3 found that the scents of women in the sexual condition were evaluated as marginally more attractive (*M* = 3.02, SD = .96) compared to the scent of the female donors in the neutral condition (*M* = 2.84, SD = .91), *F*(1, 34) = 3.99, *p* = .054, *η*^*2*^ = .11.

#### Male Recipients: Sexual Interest in the Target Women as a Function of Condition

For the first measure of sexual interest, the amount of time participants spent looking at each set of pictures, higher scores denoted relatively longer viewing times at pictures depicting promiscuous targets (computed by subtracting the time spent viewing the modest targets). In line with the hypotheses, the results found that recipients exposed to the sexual scent samples spent relatively more time looking at the promiscuous (vs. modest) targets (*M* = 6.54, SD = 9.45) than after being exposed to the neutral scents (*M* = 2.81, SD = 9.59), *F*(1, 34) = 5.31, *p* = .027, *η*^*2*^ = .14.

**Fig. 2:**
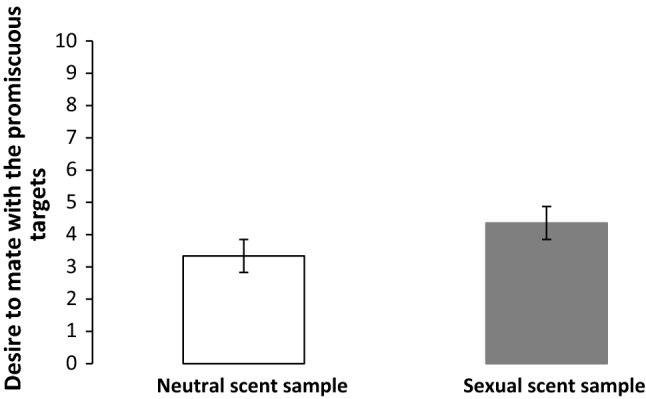
Desire to mate with the promiscuous targets after exposure to the scent samples from both conditions (Experiment 3)*. Note* Error bars represent 95% confidence intervals

Second, it was hypothesized that exposure to the sexual scent samples would increase men’s desire to mate with the promiscuous targets in particular. As predicted, it was found that participants exposed to the sexual scents reported a greater motivation to mate with the promiscuous targets (*M* = 4.36; SD = 1.36) compared to when the men were exposed to the neutral scent samples (*M* = 3.34, SD = 1.64; see Fig. 2), *F*(1, 34) = 30.81, *p* < .001, *η*^*2*^ = .48. By contrast, the scent prime condition (sexual vs. neutral) did not influence men’s mating desire toward the modest targets (*p* > .07).

Experiment 3 revealed a marginally significant finding that men judged the sexual scents as more attractive than the neutral scents. To get a clearer overall picture of the effect of the scent evaluations over three experiments, a mini meta-analysis was conducted (Goh, Hall, & Rosenthal, 2016) using fixed effects, in which the mean effect size (i.e., mean correlation) was weighted by sample size. First, partial eta-squared effect sizes were converted into Cohen’s *d* and Pearson’s correlations, as recommend by Goh et al. The correlations were then Fisher’s z transformed for the analyses. The effect was highly significant, *M r* = .37, *Z* = 3.26, *p* < .001, two-tailed, showing that men evaluated the scent of sexually aroused women as more attractive than non-sexually aroused women across all three experiments (see Table 1).

**Table 1:** Meta-analysis of within-subjects scent evaluation ratings (Experiments 1–3) as a function of scent condition (sexual vs. neutral conditions)

	*F*	*df*	*p* value	Cohen’s *d*	*r*
Study 1 (*N* = 24)	7.98	23	.010	1.18	.51
Study 2 (*N* = 32)	4.42	31	.044	.77	.36
Study 3 (*N* = 35)	3.99	34	.054	.70	.33
*M rz*					.39
*M r*					.37
Combined *Z*					3.26***

Effect sizes were calculated based on the conversion of partial eta-squared to Cohen’s *d*. Cohen’s *d* was converted into *r* (see Goh et al., 2016)

*** *p* < .001, two-tailed

The results of Experiment 3 also revealed that exposure to the sexual scents enhanced men’s subsequent sexual interest: Men spent relatively more time viewing the women displaying overt sexual cues (e.g., scantily dressed) and reported a greater desire to mate with these women. Together, these findings extend those of Experiment 2 by showing that the effects of the sexual scents altered men’s sexual motivation.

## General Discussion

The present studies provide support for the hypothesis that men are sensitive to olfactory signals of sexual arousal released by women. Overall, Experiments 1–3 and a subsequent mini meta-analysis found that men evaluated the scent of sexually aroused women as relatively more attractive. Experiment 2 showed that these sexual chemosignals increased men’s self-reported sexual arousal. Finally, Experiment 3 found support for the hypothesis that the sexual chemosignals increased men’s attention to and interest in women who displayed sexual cues. Specifically, men spent relatively more time looking at women who displayed sexual cues, and were more motivated to mate with them. Taken together, the current findings are among the first to show that that women’s sexual arousal led to the release of a distinctive scent that increases men’s sexual motivation.

These findings are consistent with numerous studies, showing that emotional states (e.g., fear, disgust, sadness) produce olfactory signals that orient nearby recipients to the immediate environment and sensitize them to emotionally consistent cues (de Groot et al., 2012; Gelstein et al., 2011; Pause, 2012; Zhou & Chen, 2008). Sexual arousal in particular is both socially and fitness-relevant states, and there are clear interpersonal benefits to its communication for both the sender and the recipient, such as the signaling and detection of mating opportunities, as well as synchronizing mating behavior between partners (Schaller, Park, & Kenrick, 2007). The current research expands on the existing literature by showing that olfactory messages may serve as an additional channel of communication between humans, and in relevant mating contexts, sexual chemosignals may be released along with corresponding visual and auditory expressions of sexual interest to produce a stronger overall signal.

Interestingly, recent research by Hoffmann (2019) also found support for the thesis that men can process the scent of sexually aroused women. Specifically, men were exposed to axillary sweat (collected from women who were sexually aroused vs. not aroused) while the men listened to erotic stories, and the findings showed that the sexual scents elicited greater genital arousal in the men. However, this effect was only detected in response to female scents collected during the luteal phase of their cycle, but not their follicular phase. In contrast to the current studies, Hoffmann (2019) did not find an effect of female scent on men’s self-reported sexual arousal and sexual interest. Those results may have diverged from the findings reported here because of several procedural differences between the two research paradigms. Notably, in our experiments, the scent samples were collected and presented to recipients under different conditions. For instance, the female scent donors in our studies briefly exercised at the start of the experiment to create a similar base rate of physiological arousal in both conditions, in order to control for physiological arousal that is also elevated during sexual arousal. Additionally, in Experiments 2 and 3, men’s sexual arousal was assessed after exposing them to a block of multiple scent samples from either scent condition, rather than each time after exposure to one scent sample. Finally, the current experiments did not present male recipients with any additional sexual stimuli (i.e., an erotic story) in conjunction with the chemosensory primes (Hoffmann, 2019). Whether or not any of these factors contributed to the different findings is an important empirical question that deserves future investigation.

Most studies have examined emotional chemosignals secreted by the axillary regions because they are dense with apocrine glands that produce sweat in response to activation of the sympathetic nervous system (de Groot, Semin, & Smeets, 2014). However, apart from perspiration, there are other volatile body fluids (e.g., urine, sperm, lacrimal fluid) that likely play roles in olfactory signaling (Pause, 2012). For instance, research has shown that exposure to scents from the vulvar area (collected during the periovulatory phase) can increase testosterone secretion and sexual interest in men (Cerda-Molina et al., 2013). In light of the current findings, it would therefore be worth testing whether women’s sexual arousal level moderates men’s responses to scents from the vulvar area.

Additionally, it would be interesting to examine the influence of the context in which men are exposed to female scents. For example, as mentioned earlier, some research paradigms have primed a sexual context when exposing recipients to the scent stimuli (Alves-Oliveira et al., 2018; Hoffmann, 2019). That is, male scent recipients listened to an erotic story or watched audiovisual stimuli (Alves-Oliveira et al., 2018) during exposure to the scents, before measuring the men’s sexual arousal. Thus, men’s reactions to the sexual scents in these studies were always a product of both the olfactory and audiovisual stimuli. In contrast, our experiments showed that the olfactory stimuli alone can elicit a sexual response in recipients, in the absence of a conceptually similar prime in a different sensory modality. Although our findings highlight that sexual chemosignals alone can prime male sexual motivation, it is unclear whether additional sexual priming via different sensory modalities can elicit stronger sexual responses in men. Thus, future research may wish to further investigate the role of priming multiple sensory modalities on how recipients are influenced by sexual chemosignals.

The current research is not without limitations. Although the indices of sexual arousal and sexual motivation used in Experiments 2 and 3 established that men respond to female chemosignals, future work would do well to examine a wider range of measurements of subjective and physiological sexual arousal (e.g., Ciardha, Attard-Johnson, & Bindemann, 2018; Janssen, Prause, & Geer, 2007; Kukkonen, Binik, Amsel, & Carrier, 2007; Laws, 2009; McPhail et al., 2019). In addition, while our studies did not take the donors’ menstrual cycle into account, the recent findings of Hoffmann (2019) highlight that there is scope to further investigate the interaction between menstrual cycle phase and women’s axillary chemosignals, and the influence of these signals on male sexual arousal (see Hoffmann, 2019, for a full discussion of the results). Additionally, future research in chemosignal research would benefit from considering procedural differences in order to understand which factors tend to enhance and mitigate the effects of sexual chemosignals on recipients (Pause, 2012). Moreover, it is perhaps worth considering how sexual arousal chemosignals interact with individual factors we did not specifically examine, such as testosterone levels (Gangestad, Thornhill, & Garver-Apgar, 2010; Thornhill & Gangestad, 1999), or individual differences in disgust sensitivity (Haidt, McCauley, & Rozin, 1994; Stevenson, Case, & Oaten, 2011). Finally, future work could include a wider range of measures to monitor the emotions of the scent donors and the scent recipients during the experiment (de Groot et al., 2015b; Mitchell, DiBartolo, Brown, & Barlow, 1998).

Consistent with the growing evidence that emotional states can be communicated through scent, our findings provide evidence that humans can signal and process olfactory signals of sexual arousal. Importantly, the results showed that perceiving these sexual chemosignals alters the scent receiver’s sexual arousal and their interest and preference for potential mates. Informed by the present findings, we can envision a dynamic exchange of olfactory signals that, combined with corresponding visual and auditory expressions, are communicated between men and women during mating encounters. These encounters may thus entail more than meets the eye and we hope that the current findings encourage further research to examine the role of sexual olfactory signals in human communication.

## Footnotes

1 The sample size of the scent recipients was determined a priori using G*Power (Faul, Erdfelder, Buchner, & Lang, 2009) based on a within-subjects design (allowing for mixed-model analyses in Experiments 2 and 3) and a medium effect size *f* of .35 (Cohen, 1992; Lakens, 2013) to obtain a power of .95. The actual effect sizes of the main effects were between medium and large (see Table 1).

2 One participant did not meet the inclusion criteria (i.e., about not taking any form of chemical contraception) and was therefore excluded from the final sample of scent donors.

3 Note that the results of Experiments 2 and 3 were not altered when the order in which the scent blocks (sexual versus neutral) were presented was analyzed as a separate factor.

4 The pilot study asked sixty males (*M*_Age_ = 26.60; SD_Age_ = 2.53) to evaluate the photographs of 20 target women, in which ten were preselected as being high in sexual salience and ten were low in sexual salience. The order in which the target photographs were presented was randomized, and the two groups of target photographs were not distinguished or labeled in any way. Each woman was rated in terms of how good-looking she was (“How good looking is this person?”; 1 = *not at all* and 10 = *very much*), how revealingly she was dressed (“Is this person dressed in a revealing way?”; 1 = *not at all* and 10 = *very much*), and how promiscuous she appeared to be (“Would it be easy to have sex with this person?”; 1 = *not at all* and 10 = *very much*). The results showed that the targets high in sexual salience were rated as less good-looking (*M* = 5.68; SD = 1.88) than the targets low in sexual salience (*M *= 7.22; SD = 1.90; *p* < .001); the targets high in sexual salience were also judged to be dressed more revealingly (*M *= 7.31; SD = 2.19) than the women that were low in sexual salience (*M* = 2.53; SD = 1.93; *p* < .001); and the targets high in sexual salience were judged to be more promiscuous (*M* = 5.79; SD = 2.34) than the targets low in sexual salience (*M* = 4.69; SD = 2.56; *p* < .001).
